# Functional difficulty among young children in Bangladesh: An analysis of nationally representative data

**DOI:** 10.1371/journal.pone.0300403

**Published:** 2024-03-21

**Authors:** Maisha Maliha Rahman, M. Iftakhar Alam, Mohaimen Mansur

**Affiliations:** Institute of Statistical Research and Training, University of Dhaka, Dhaka, Bangladesh; Jahangirnagar University, BANGLADESH

## Abstract

Functional difficulty in children is a crucial public health problem still undervalued in developing countries. This study explored the socio-demographic factors and anthropometry associated with children’s functional difficulty in Bangladesh. Data for 2-4-year-old children, obtained from Multiple Indicator Cluster Survey 2019, were used in this study. The mixed-effects logistic regression model was used to analyse the data. Children whose mothers had functional difficulty were found to be 2.75 times more likely to have functional difficulty than children whose mothers had no functional difficulty (95% CI 1.63-4.63). Male children were more likely to experience functional difficulty than female children (OR = 1.48). Furthermore, stunting was found to be significantly associated with functional difficulty (OR = 1.50). The study also revealed that division and mother’s education, specifically, children with mothers having higher secondary + education, had significant association with the outcome variable. The findings provided a vital overview of child disability in a developing country.

## Introduction

Child functional difficulty has been a persistent problem attracting growing interest from public health specialists worldwide. Such difficulties can range from impairments such as hearing and vision loss, epilepsy, cerebral palsy, attention deficit hyperactivity disorder, autism spectrum disorder, intellectual disability, or other learning disorders, with their chronic adverse effects perpetuating into adulthood [1–4]. Childhood disability has an adverse impact on education and employment. The prevalence of children with disabilities in a country varies noticeably depending on the methodology used to measure disability [5]. To ensure reliable data, the Child Functioning Module (CFM) has been developed by the Washington Group on Disability Statistics and UNICEF [6, 7].

“Leave no one behind” is a primary objective of Sustainable Development Goals (SDGs) [8]. It directly recognises the importance of addressing the needs of people with disabilities along with other vulnerable groups of society. Following the finalisation of CFM in 2016, a joint statement was issued in 2017 by multiple UN agencies, member states, organisations of persons with disabilities, and other stakeholders, recommending the CFM as an appropriate tool for SDGs data disaggregation for children. The module has been included in the most recent UNICEF-supported Multiple Indicator Cluster Surveys (MICS-6). The intention was to identify the children more likely to encounter participation limitations in an environment that does not accommodate functional difficulties. The limited participation of children due to disability was investigated by several authors. For example, a consistent and statistically significant disability gap in primary and secondary school attendance had been found in an investigation of fifteen low-and middle-income countries [9]. Childhood disability had been found to be associated with an increased probability of individuals falling in the poorest wealth quintile [10]. The disability may even increase violent behaviour against children [11]. Increased levels of depression and anxiety symptoms were found amongst parents with a child with intellectual and developmental disabilities [12]. Hamdani et al. [13] validated the World Health Organisation’s disability assessment schedule for children in Pakistan. Chen et al. [12] conducted a systematic review that synthesised measures assessing activities of daily living for children with developmental disabilities.

Bangladesh is a lower-middle-income country, and about 10% of the population is children under five years old [14]. It was among the first countries to corroborate and bring into force the two most remarkable global agreements that protect the rights of children with disabilities: the Convention on the Rights of the Child (1990) and the Convention on the Rights of Persons with Disabilities (2007) [15]. The Convention on the Rights of Persons with Disabilities outlines states parties’ obligations to ensure the full realisation of rights for children with disabilities on an equal basis with other children [16]. The CFM has been utilised for the first time in Bangladesh in MICS 2019. This paper aims to utilise this recently available dataset to provide an overview of childhood functional difficulties in Bangladesh and determine important socio-demographic factors associated with these difficulties. Identifying the factors is essential so that necessary measures can be taken to minimise the problems of children with functional difficulties in the country. Moreover, these would help design policies and interventions to ensure such children’s full inclusion and participation in society.

## Materials and methods

### Ethics approval

The survey protocol was approved by the technical committee of the Government of Bangladesh, led by the Bangladesh Bureau of Statistics. It would be useful to mention that this paper does not involve any data collected by the authors. Verbal consent was obtained for each respondent participating in the survey. All respondents were informed of the voluntary nature of participation and the confidentiality and anonymity of information. Additionally, respondents were informed of their right to refuse to answer all or particular questions and stop the interview at any time. It would be useful to mention that the authors did not have access to information that could identify individual participants during or after data collection.

### Data

The source of the data for this study was MICS 2019, which the Bangladesh Bureau of Statistics conducted in cooperation with UNICEF Bangladesh [17]. The survey was designed to provide national-level estimates for many indicators on the situation of children and women in the eight regional divisions and sixty-four districts of the country. A two-stage stratified cluster sampling design was used to collect data. The districts were identified as the main sampling strata, and the sample of households was selected in two stages. A specified number of census enumeration areas within each stratum were selected systematically with probability proportional to size. After a household listing was carried out within the selected enumeration areas, a systematic sample of 20 households was drawn in each selected enumeration area. The data obtained from the survey were released in March 2020. The dataset used in this study was the “ch” dataset (children under 5 dataset), from which information on children aged 2-4 years was extracted as there was no information on disability for children under 2 years old. We referred to these 2- to 4-year-old children as “young children” in this paper.

### Response variable

The “ch” dataset contained information on all the variables included in the CFM. The primary response variable of interest in this study was the functional difficulty of 2- to 4-year-old young children. There were eight domains of functional difficulty for them: seeing, hearing, walking, fine motor, communication, learning, playing and controlling behaviour. There were four response options for each of the first seven domains: no difficulty, some difficulty, a lot of difficulty and cannot do it at all. Suppose the mother or primary caregiver responded to “a lot of difficulty” or “cannot do it at all” to the questions for a domain. In that case, a child is considered to have functional difficulty in that domain [17, 18]. There were five response options for the last domain, controlling behaviour: not at all, less, the same, more and a lot more. The response to “a lot more” was considered a functional difficulty in this domain [17]. If a 2-4-year-old child had difficulty in at least one of these eight domains, the child had functional difficulty, the response variable of interest. Note that this response variable was binary and available in the dataset as *cdisability* (“has no functional difficulty”, “has functional difficulty”). Indeed, the response variable was a composite indicator of disability in the sense that its value depended on the responses of various domains.

### Explanatory variables

The explanatory variables used in this study were sex (“female”, “male”), area of residence (“rural”, “urban”), division (“Barishal”, “Chattogram”, “Dhaka”, “Khulna”, “Mymensingh”, “Rajshahi”, “Rangpur”, “Sylhet”), age (“2”, “3”, “4”), mother’s education (“pre-primary or none”, “primary”, “secondary”, “higher secondary+”), mother’s functional difficulty (“no”, “yes”), wealth index quintile (“poorest”, “second”, “middle”, “fourth”, “richest”) and stunting (“no”, “yes”). Children with stunting were defined as those who fall under minus two standard deviations (moderate and severe) of the median height-for-age, as recommended by the World Health Organization [19].

For collecting data on mothers’ functional difficulty, MICS 2019 utilised an adult functioning module developed by The Washington Group on Disability Statistics [20]. The questions in the module reflected six domains for measuring disability: seeing, hearing, walking, cognition, self-care and communication. Each question had four response categories: no difficulty, some difficulty, a lot of difficulty and cannot do it all. A woman was considered to have functional difficulty in a domain if she was experiencing at least “a lot of difficulty” in that particular domain. For each woman, the overall binary disability indicator “functional difficulty” was defined as someone who reported to have a lot of difficulty or unable in at least one domain. The “mother’s functional difficulty” contained information for the mother of the concerned child and was available in the MICS 2019 dataset. It would be useful to mention that the adult functioning module was found reliable to measure disability in a study in fifteen countries of Central and South America, Asia and Africa [21].

The wealth index was a composite measure of the living standard of a household, which was calculated using the principal component analysis of information on the ownership of consumer goods, dwelling characteristics, water and sanitation, and other related characteristics to the household’s wealth. All the participating households in the survey were classified into quintile groups based on their principal component scores [17, 22].

The divisions used in this study were broad geographic classifications and may vary in terms of overall poverty, literacy rate, public health conditions, cultural practices, urbanity-rurality proportions, etc. These division-level aggregate information were not available in the dataset but including divisions in the analysis controls for unmeasured or unobserved spatial variations that may influence child functionality.

### Statistical analysis

Since the response variable was binary with the presence or absence of any functional difficulty as categories, the bivariate and multivariate analyses were performed to identify the influential socio-demographic covariates. The bivariate analysis reported the percentages of response for each covariate category and the *P*-values for the chi-square tests [23] of a possible association between the two variables. MICS data had a hierarchy as they came from two-stage sampling. It was natural for children from the same cluster to have some common features, leading to correlated responses within a cluster. Using a fixed-effects logistic regression model was not a good choice since it fails to quantify the variability due to clusters. Also, such a model provides incorrect standard error of the estimates [24]. We therefore used a mixed-effects logistic regression model for our data [25] to overcome these issues.

The binary response that *j*th child in *i*th cluster had functional difficulty was represented by *Y*_*ij*_ and let *π*_*ij*_ be the probability of that event. The set *X* = {*X*_1*ij*_, *X*_2*ij*_, …, *X*_*kij*_} represented the *k* observed variates for *j*th child in *i*th cluster. The following model was then fitted to the data

$$\begin{array}{c} \text{log}(\frac{\pi_{ij}}{1-\pi_{ij}})=\beta_{0}+\beta_{1}X_{1ij}+\beta_{2}X_{2ij}+\ldots +\beta_{k}X_{kij}+u_{i}, \end{array}$$

where *β*_*k*_ was the coefficient corresponding to *X*_*k*_ and *u*_*i*_ was the random effect associated with *i*th cluster. It would be useful to mention that the considered model was a random intercept model. The underlying assumption was that *u*_*i*_s had a normal distribution with mean 0 and constant variance $\sigma_{u}^{2}$. The *β*_*k*_ reflected the effect of covariate *X*_*k*_ averaged over the clusters. The between-cluster variance and within-cluster variance were used to obtain the intra-cluster correlation (ICC) as [24]

$$\begin{array}{c} \rho =\frac{\sigma_{u}^{2}}{\sigma_{u}^{2}+\frac{\pi^{2}}{3}}. \end{array}$$

If *ρ* was close to zero, there would be no variation in responses due to clusters; alternatively, if *ρ* > 0, there would be variation in responses due to clusters. Survey weights weighted the data before analysis since they were not self-weighted, and the analyses were performed using Stata [26].

## Results

This section presents the numerical results obtained from the bivariate and multivariate analyses for young children aged 2-4 years. A total of 14,072 children were available, and 394 of them (2.8%) were found to have functional difficulty. The sections below give further details.

### Bivariate analysis

Table 1 shows the prevalence of functional difficulty in percentages at various levels of the covariates for each of the domains of difficulty. The proportion of children under a composite indicator of disability defined by functional difficulty in at least one of the individual domains is also reported in the column prior to the last one. *P*-values presented in the last column indicate whether explanatory variables are statistically significantly associated with the composite disability indicator under the chi-square test.

**Table 1:** Percentage of children aged 2-4 years who had functional difficulty, by domains in Bangladesh, 2019.

Characteristic	Seeeing	Hearing	Walking	Fine motor	Communication	Learning	Playing	Controlling behaviour	Functional difficulty in at least one domain	*P*-value (*χ*^2^ test)
Total	0.1	0.2	0.4	0.4	0.7	1.2	0.5	1.1	2.8	
Sex										
Male	0.1	0.1	0.4	0.4	8.0	1.2	0.6	1.3	3.2	0.001
Female	0.1	0.2	0.3	0.3	0.5	1.1	0.3	0.9	2.3	
Area										
Urban	0.2	0.1	0.2	0.5	0.5	0.8	0.3	1.8	3.3	0.077
Rural	0.1	0.2	0.4	0.4	0.7	1.3	0.5	0.9	2.7	
Division										
Barishal	0.4	0.4	0.3	0.2	1.6	6.8	1.2	0.7	8.5	<0.001
Chattogram	0.2	0.1	0.2	0.2	0.5	0.7	0.4	0.2	1.3	
Dhaka	0.0	0.1	0.2	0.2	0.7	0.6	0.3	3.0	4.1	
Khulna	0.2	0.2	0.3	0.1	0.3	0.2	0.4	0.5	1.5	
Mymensingh	0.1	0.1	0.7	0.7	1.0	3.3	0.8	1.9	5.8	
Rajshahi	0.3	0.3	0.3	0.4	0.7	0.6	0.6	0.3	1.5	
Rangpur	0.2	0.3	0.4	0.5	0.6	0.6	0.4	0.8	1.7	
Sylhet	0.3	0.2	0.6	0.6	0.7	0.9	0.6	0.2	1.3	
Age										
2	0.2	0.1	0.4	0.4	0.8	1.3	0.6	0.8	3.0	0.501
3	0.1	0.2	0.4	0.4	0.6	1.0	0.4	1.4	2.8	
4	0.1	0.1	0.2	0.2	0.5	1.2	0.4	1.2	2.6	
Mother’s education										
Pre-primary or none	0.4	0.3	0.5	0.5	1.1	2.3	0.6	1.3	4.1	0.001
Primary	0.2	0.2	0.3	0.3	0.6	1.3	0.6	1.0	2.8	
Secondary	0.1	0.1	0.3	0.3	0.7	1.0	0.3	1.1	2.6	
Higher secondary+	0.1	0.0	0.1	0.3	0.1	0.7	0.2	1.0	2.1	
Mother’s func. diffi.										
Yes	0.9	0.5	0.5	0.9	2.3	5.0	0.5	2.3	10.4	<0.001
No	0.1	0.2	0.2	0.3	0.6	1.1	0.4	1.1	2.6	
Wealth index quintile										
Poorest	0.3	0.2	0.5	0.3	0.7	1.9	0.5	0.9	3.3	0.024
Second	0.2	0.3	0.4	0.4	0.8	1.7	0.8	1.0	3.3	
Middle	0.0	0.1	0.2	0.1	0.5	0.9	0.3	1.0	2.1	
Fourth	0.2	0.2	0.3	0.5	0.8	0.8	0.4	1.0	2.6	
Richest	0.1	0.1	0.1	0.3	0.4	0.5	0.2	1.6	2.5	
Stunting										
Yes	0.0	0.2	0.5	0.3	0.6	1.7	0.5	1.2	3.5	<0.001
No	0.1	0.0	0.1	0.1	0.4	0.7	0.1	1.1	2.2	

Around 3% of the young children experienced at least one of the functional difficulties. While learning and controlling behaviour difficulties were the most prevalent among sample children (1.2% and 1.1%, respectively), only small percentages experienced difficulties in seeing and hearing (0.1% and 0.2%, respectively). Sex had a significant association with the composite disability. Male children had 0.9 percentage points higher prevalence of overall disability than female children. A closer look at the individual domains of difficulties showed that the prevalence of difficulties in communication among male children was prominently 7.5 percentage points higher than that in female children. Other notably higher prevalences of difficulties in boys was observed in controlling behaviour (0.4 percentage points) and playing (0.3 percentage points). Gender gaps in the prevalence of the remaining types of difficulties were marginal. There was no statistically significant association between age and overall functional difficulty. Difficulties were lower at higher ages except for difficulties in hearing, learning and controlling behaviour. For example, the proportion of boys with communication difficulties was 0.3 percentage points lower in 4-year-old children than in 2-year-old children. Conversely but prominently, 0.6 and 0.4 percentage points higher prevalences of difficulties in controlling behaviour were observed in 3-year and 4-year-old children, respectively, compared to younger children of 2 years of age.

When the area of residence was concerned, the overall disability index was 0.6 percentage points lower in rural children, but this difference with urban children was not statistically significant. Rural children had a substantially lower prevalence of difficulty (0.8 percentage points) in controlling behaviour than urban children and marginally lower prevalences of 1 percentage point in each of seeing and fine motor. In other domains of difficulties, rural children had higher prevalences of difficulties. In particular, children living in rural areas had a much higher prevalence of difficulty in learning (1.3%) than their urban counterparts (0.8%). Also, marginally more children (2 percentage points higher prevalence) experienced difficulty in walking, communication and playing in rural areas. Rural-urban differences in the prevalence of functional difficulties in other domains were minimal.

There was statistically significant variation in the prevalence of children having functional difficulties in at least one of the domains across the eight administrative divisions of the country. The highest prevalences under this composite disability could be observed in Barishal (8.5%), Mymensingh (5.8%) and Dhaka (4.1%), with prevalences in the rest of the divisions below 2%. Prevalences of seeing and hearing difficulties across divisions ranged from 0%-4% with the highest percentages in Barishal and lowest in Dhaka and Mymensingh.

Percentages of children experiencing difficulties in walking and fine motor ranged from 1%-7%, and these difficulties were more prevalent in Mymensingh and Sylhet and less prevalent in Dhaka, Chattogram and Khulna division. Prevalence of learning difficulties was extremely high (6.8%) in Barishal relative to other divisions. The second highest prevalence of disability of 3.3% in this domain was observed in Mymensingh, while the rest of the divisions had prevalences below 1%, with Khulna recording the lowest (0.2%). Barishal was also the only division with above 1% prevalences of difficulties in communication (1.6%) and playing (1.2%). The percentage of children with difficulties in controlling behaviour was the highest in Dhaka (3%) and was also substantially higher in Mymensingh (1.9%) compared to other divisions. Khulna division had low prevalences of functional difficulties across all the domains, with disability percentages not exceeding 0.5%.

Mother’s education was negatively, yet statistically significant, associated with the composite indicator of disability, and domain-specific functional difficulties in children were typically lower at higher levels of mother’s education. Differences in the prevalences of child disabilities in the highest and lowest levels of maternal education were particularly notable in learning and communication. More precisely, compared to children of mothers with no education or just pre-primary education, children of mothers with more than higher secondary education had a 1.6 percentage points and 1 percentage point lower prevalences of difficulties in learning and communication, respectively. Such differences were, however, small in other domains of difficulties, including physical, fine motor and behavioural disabilities and ranged between 0.1-0.4 percentage points.

Children of mothers who themselves experienced some form of functional difficulties had higher prevalences of functional difficulties in all domains of disabilities than children whose mothers did not face any such difficulties. The differences in prevalences of various types of child disability for the two groups of mothers ranged from 0.1-3.9 percentage points, with the largest margins of differences observed in cases of difficulties in learning (3.9 percentage points), communication (1.7 percentage points) and controlling behaviour (1.2 percentage points). According to the composite indicator of disability, the prevalence of functional difficulty in at least one of the domains in children of mothers with disabilities (10.4%) was four times the prevalence in children with mothers not facing such difficulties. This association between children’s and mothers’ disabilities were strongly significant, with a *P*-value below 0.001.

There was also a statistically significant relationship between household wealth and composite indicator of disability. The prevalence of child disability was lower in households belonging to the highest wealth quintile than in households with the lowest level of wealth, except in controlling behaviour. The largest difference in prevalence between these two extreme wealth groups was observed in learning (1.4 percentage points). In other domains, gaps varied between 0.0-0.4 percentage points. For controlling behaviour, an inverse pattern was observed: children from the richest households had a 0.7 percentage points higher prevalence of functional difficulties than children from the poorest households. Interestingly, disability percentages were not always consistently lower at subsequent levels of higher household wealth. For example, in the case of communication difficulties, the prevalence of difficulties was the lowest in the highest wealth quintile but the highest in the second and fourth wealth quintiles. Also, the prevalence of difficulty in the middle wealth quintile was the lowest in certain instances, e.g., fine motor and controlling behaviour, leading to the lowest overall composite disability in the group.

Stunting (low height-for-age), a malnutrition outcome, was positively and statistically significantly associated with overall child disability. Prevalences of different types of disabilities were higher in stunted children in comparison to non-stunted children, except for seeing difficulties. These differences ranged from 0.1-1 percentage points, with larger differences observed for learning, playing and walking difficulties.

### Multivariate analysis

Table 2 shows the mixed-effects logistic regression analysis results considering child functional difficulty in at least one domain as the response. Regression estimates and their standard errors (SE) were reported together with associated *P*-values, odds ratio (OR) and 95% confidence intervals (CI). Sex had a significant association with the response variable (*P* < 0.01), confirming the finding from the bivariate analysis. Male children were 48% more likely to have functional difficulty than female children (OR = 1.48). The area of residence was not a significant predictor of the outcome variable (*P* = 0.54). However, the odds of functional difficulty varied significantly across regional divisions in the country except for Mymensingh, where the association was insignificant. Children of Chattogram, Dhaka, Khulna, Rajshahi, Rangpur and Sylhet was 87%, 0.62%, 77%, 85%, 79% and 92% less likely to have functional difficulty compared to children from Barishal, respectively. Children in Barishal were more likely to have functional difficulty among all the divisions. Age had no significant relationship with the occurrence of functional difficulty.

**Table 2:** Results of mixed-effects logistic regression for functional difficulty in children aged 2-4 in Bangladesh, 2019.

Characteristic	Coefficient	SE	*P*-value	OR	95% CI for OR
Sex (Ref: Female)					
Male	0.39	0.12	<0.01	1.48	1.16, 1.87
Area (Ref: Rural)					
Urban	0.11	0.18	0.54	1.11	0.79, 1.58
Division (Ref: Barishal)					
Chattogram	-2.02	0.54	<0.01	0.13	0.05, 0.39
Dhaka	-0.98	0.54	0.05	0.38	0.14, 1.00
Khulna	-1.46	0.54	0.01	0.23	0.08, 0.67
Mymensingh	-0.41	0.64	0.52	0.66	0.19, 2.33
Rajshahi	-1.93	0.60	<0.01	0.15	0.05, 0.47
Rangpur	-1.58	0.57	0.01	0.21	0.07, 0.63
Sylhet	-2.58	0.75	<0.01	0.08	0.02, 0.33
Age (Ref: 2)					
3	-0.05	0.14	0.79	0.95	0.72, 1.25
4	-0.05	0.15	0.69	0.95	0.71, 1.27
Mother’s education (Ref: Pre-primary or none)					
Primary	-0.29	0.19	0.12	0.74	0.51, 1.08
Secondary	-0.34	0.19	0.06	0.71	0.49, 1.02
Higher secondary+	-0.65	0.26	0.01	0.52	0.31, 0.87
Mother’s functional difficulty (Ref: No)					
Yes	1.01	0.27	<0.01	2.75	1.63, 4.63
Wealth index quintile (Ref: Poorest)					
Second	0.14	0.17	0.40	1.15	0.83, 1.60
Middle	-0.10	0.20	0.61	0.90	0.61, 1.33
Fourth	0.09	0.21	0.65	1.10	0.73, 1.65
Richest	0.09	0.26	0.73	0.91	0.55, 1.52
Stunting (Ref: No)					
Yes	0.40	0.13	<0.01	1.50	1.17, 1.91
Constant	-3.04	0.46	<0.01	0.90	0.02, 0.12

Children with mothers whose education level was higher secondary + are 48% less likely to have functional difficulty than children with mothers whose education level was pre-primary or none (OR = 0.52). Children born to mothers with functional difficulty were 2.75 times more likely to have functional difficulty themselves than children born to mothers with no difficulty. Wealth index quintile did not appear to be significantly related to child functional difficulty. Stunting had a significant positive association (*P* < 0.01) with an odds ratio of 1.50, i.e., stunted children were 50% more likely to experience functional difficulty than non-stunted children. The estimate of $\sigma_{u}^{2}$ was found to be 0.90, which resulted in the intra-cluster correlation estimate as 0.20. That is to say, 20% of the variation in the response variable was due to the variation in clusters.

### Further results

While the mother’s education and wealth index quintile were statistically significantly associated with the composite disability indicator in the bivariate analysis, the wealth index appeared insignificant in the multivariate analysis and maternal education was significant in multivariate analysis only at the highest level. In order to understand these contradictory results, we further explored the possible bivariate association between maternal education and wealth index, and each of these two variables with mother’s functional difficulty, which was the most dominant predictor of functional difficulty of children in our analysis. Table 3 shows that all the bivariate associations were strongly significant (*P* < 0.001).

**Table 3:** Association between selected explanatory variables.

Variables	*P*-value (*χ*^2^ test)
Mother’s education, wealth index quintile	<0.001
Mother’s education, mother’s functional difficulty	<0.001
Wealth index quintile, mother’s functional difficulty	<0.001

## Discussion

This study attempted to provide an overview of the functional difficulty of young children in Bangladesh. Following standard guidelines adopted in MICS 2019, several physical, cognitive and social-emotional domains were considered for defining child functionality. A child is considered to have functional difficulty when s/he has failed to sufficiently cope with any of the difficulties, as reported by the mother or primary caregiver.

Sex appeared to have a significant association with child functioning, and male children seemed to be more likely to experience difficulties than female children. The prevalence of a child’s functioning varied significantly across the divisions. Children in almost all divisions had substantially lower chances of having functional difficulty than those in Barishal. Notice that children in the Sylhet division had the least likelihood of experiencing functional difficulty.

According to the findings of this study, mothers’ experience of their functional difficulties was by far the most prominent predictor of functional difficulties in their children. Risks of experiencing functional difficulties were almost three-fold higher in children of mothers with functional difficulties than in children with mothers having no such difficulties. This observed association was in line with the findings of another study on childhood disability using MICS data [27]. The significant role of mother’s disabilities on poor early childhood cognitive development was also reported in another MICS-based study on Bangladesh [28]. Managing child behaviour, controlling them or setting boundaries and teaching them properly can be very challenging for a mother with functional difficulty [29], which may lead to behavioural difficulties among children. Also, there is increasing evidence that certain physical and mental disabilities such as visual impairment, hearing loss, and depression can be genetically inherited by off-springs from parents [30–34]. However, whether these potential causal mechanisms played a role in the mother-child disability relationship can not be verified by the data used in this study.

The highest level of a mother’s education was found to have a significant association with lower functional difficulty. Such a negative relationship between maternal education and childhood disability is also evident in the literature [35]. However, no significant association had been found between wealth index quintile and functioning difficulty while doing multivariate analysis, even though it was observed during bivariate analysis. A deeper investigation of the data showed that the mother’s functional difficulty, the most dominant predictor of a child’s functional difficulty, had a strong statistically significant bivariate association with wealth status. This close relationship may have concealed the direct influence of financial condition on a child’s functional difficulty after controlling for the mother’s functional difficulty and explains the non-significant association in the multivariate setting. Stunted children appeared to be more likely to have functional difficulties than non-stunted children. Stunting reflects the aggregate repercussions of undernutrition in children since birth or even prenatal stages, indicating long-term restriction on a child’s growth potential [36]. Although this study does not establish a causal connection between disability and malnutrition, it confirms the importance of childhood under-nutrition in explaining part of variations in young children’s risks of experiencing functional difficulties in Bangladesh. Although a study similar to this paper was conducted by Anjum et al. [37], their set of explanatory variables is slightly different. More importantly, they ignored the hierarchical nature of data and fitted a fixed-effects logistic regression model.

## Strengths and limitations

The data utilised in this study are nationally representative data collected through the MICS 2019. Therefore, the results presented in the paper provide a reliable summary of the functional difficulties of children in Bangladesh. This study also considers the nutritional statuses of children aged 2 to 4 as potential determinants of childhood disability, along with demographic and socio-economic factors. Taking all these different spectrums can help policymakers to design relevant policies and interventions.

However, this study still has a few limitations. First, this study investigated the association between the functional difficulty of children and its potential socio-demographic risk factors using cross-sectional data. Thus, the nature of the data and study does not allow us to make any causal inference. Second, the respondents of the questionnaires for children aged 2-4 are their mothers who have different socio-economic backgrounds. While the questions in the CFM were tested in different countries, differences in how they were interpreted and the accuracy of responses across different socio-economic characteristics of the respondents may still affect, possibly not by large margins, the relationships between the covariates and functional difficulty found in this paper. Third, while creating a composite indicator of disability is useful for summarising the data and understanding the prevalence of disability and its relationship with other covariates, a more detailed analysis would be needed to understand the full complexity of diverse functional difficulties and how they arise and develop.

## Conclusion

This paper provides an overview of the situation of functional difficulty of young children in Bangladesh. Regarding socio-demographic determinants, administrative divisions of residence and the mother’s functional difficulty appear to be the essential determinants of the functional difficulty of children in the country. Height-for-age (stunting), an outcome of growth limitation due to malnutrition, is also a significant predictor of functional difficulties in children 2-4 years of age. Since children with functional difficulties are known to have highly negative future consequences in life, it is necessary to support these children from an early age to reach development potential for active participation in society. Policies targeted to improve young children’s nutritional status and provide support to mothers with difficulties may help children with functional difficulties overcome their difficulties.
